# Mizoribine as a safe and effective combined maintenance therapy with prednisolone for anti-neutrophil cytoplasmic antibody-associated vasculitis in a hemodialysis patient

**DOI:** 10.1007/s13730-012-0050-1

**Published:** 2013-01-30

**Authors:** Gen Nakamura, Noriyuki Homma, Yuichi Sakamaki, Mio Toyama, Megumi Unno, Takeshi Kuroda, Ichiei Narita

**Affiliations:** 1 Division of Nephrology, Niigata Prefectural Shibata Hospital, 2-8, Motomachi 1-chome, Shibata, Niigata 957-8588 Japan; 2 Division of Clinical Nephrology and Rheumatology, Niigata University Graduate School of Medical and Dental Sciences, 1-757 Asahimachi-dori, Cyuuou-ku, Niigata, 951-8510 Japan

**Keywords:** Mizoribine, Hemodialysis, Pulmonary hemorrhage, Proteinase 3 anti-neutrophil cytoplasmic antibody-associated vasculitis

## Abstract

A 77-year-old man developed severe renal insufficiency due to proteinase 3 anti-neutrophil cytoplasmic antibody (PR3-ANCA)-associated vasculitis, and was started on hemodialysis (HD). Because his renal insufficiency appeared to be irreversible, he was maintained on oral prednisolone (PSL) at 5 mg/day. However, a disease flare-up with alveolar hemorrhage occurred. Serology revealed elevated levels of PR3-ANCA and C-reactive protein (CRP). The patient was given pulse therapy with a quarter dose of methylprednisolone (m-PSL) (250 mg, 3 days), followed by oral PSL at 15 mg/day. As a supplemental treatment, he was given 25 mg of mizoribine (MZR) immediately after each HD session. Subsequently, the levels of PR3-ANCA and CRP decreased, and the alveolar hemorrhage resolved. The dose of MZR to be given was determined by measuring the patient’s serum concentrations of MZR at various time points after the HD session. The maintenance dose of MZR was finally set at 50 mg. At present, the oral PSL dosage has been tapered to 10 mg/day, and the patient has achieved a state of remission without any side effects.

## Introduction

Since combination therapy with steroids and cyclophosphamide was first introduced, the prognosis for patients with anti--neutrophil cytoplasmic antibody (ANCA)-associated vasculitis has improved significantly [1, 2]. However, the effectiveness of this therapy has proved to be limited, and adverse reactions to these medications can occur, especially in patients with renal failure. In addition, we have frequently experienced relapses of the disease activity in patients undergoing a tapering of the prednisolone (PSL) dosage, even after the induction of disease remission. It is important for these patients to maintain their disease in remission mode as long as possible without adverse effects. For these reasons, a combination of steroids and immunosuppressive drugs such as mizoribine (MZR) has been used in order to improve the effectiveness of the steroids and to reduce adverse reactions to them.

MZR is an imidazole nucleoside [3] that blocks purine biosynthesis [4, 5]. MZR inhibits both humoral and cellular immunity by selectively blocking the proliferation of lymphocytes [6]. Its clinical efficacy was first demonstrated in renal transplant recipients [7], and controlled trials conducted in Japan demonstrated that this drug prolonged graft survival without myelosuppression or hepatotoxicity [8]. MZR’s immunosuppressive effect is equivalent to that of azathioprine, but it is safer than other immunosuppressive drugs [8, 9]. Hyperuricemia is the most common adverse event associated with MZR (16 %), but it is transient. MZR has been used to prevent renal transplant rejection [10] and to treat rheumatoid arthritis [11], nephritic syndrome [12], IgA nephropathy [13], and systemic lupus erythematosus [14].

Recent reports have shown MZR to be safe and effective, even against ANCA-associated vasculitis in patients without renal insufficiency or with mild renal insufficiency [15–17]. MZR is mainly excreted from the kidneys, and its plasma concentration depends greatly on renal function [18]. Therefore, in patients with renal insufficiency, it is necessary to monitor the serum concentration of MZR to prevent adverse drug-related effects.

We herein report the case of a HD patient with proteinase 3-ANCA (PR3-ANCA) associated vasculitis, in whom combined therapy with MZR and PSL successfully elicited the remission of alveolar hemorrhage, lowered the PR3-ANCA titer, and maintained disease remission.

## Case report

**Fig. 1:**
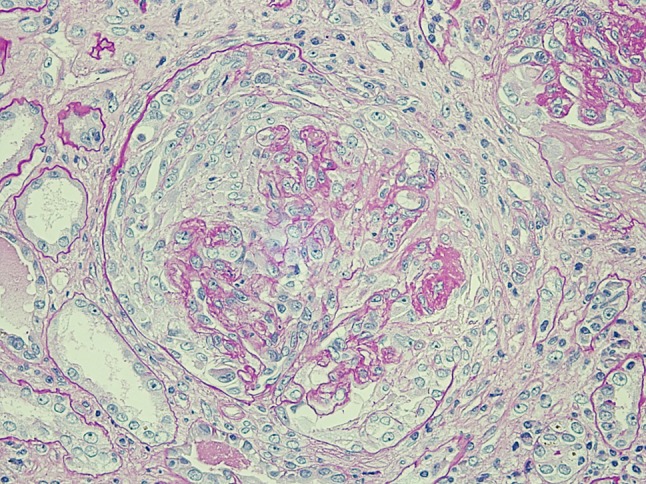
Renal biopsy specimen in the current case. Light microscopic examination showed cellular crescents in 10 of 13 glomeruli (periodic acid-Schiff stain)

**Fig. 2:**
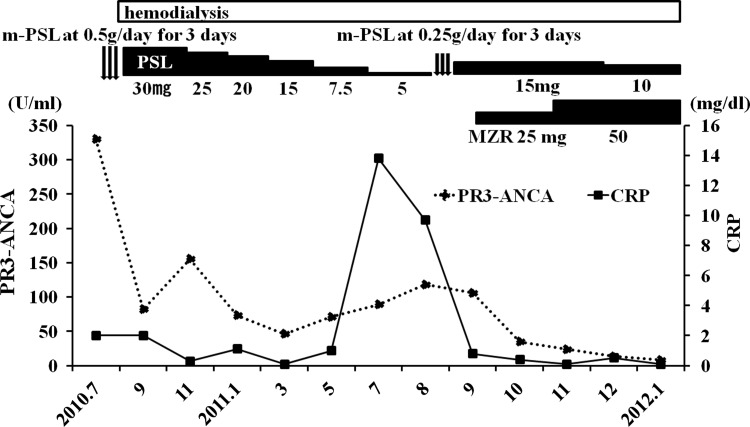
Time courses of the patient’s PR3-ANCA and CRP levels. The levels decreased after induction therapy with m-PSL and oral PSL. However, the ANCA-associated vasculitis flared up after the PSL dose was decreased to 5 mg/day. The patient was then readmitted in July 2011. BALF analysis confirmed alveolar hemorrhage. m-PSL was administered at 250 mg/day for 3 days, followed by oral PSL at 15 mg/day. Two weeks later, a supplemental treatment of 25 mg MZR administered immediately after each HD session was initiated. The maintenance dose of MZR was finally set at 50 mg, after monitoring its serum concentration. These treatments relieved the patient’s symptoms, and the CT findings disappeared completely

A 77-year-old Japanese man complaining of fever, general fatigue, and appetite loss was admitted to our hospital in July 2010. He had suffered from leg edema for five months, which had worsened over time. He was treated for chronic obstructive pulmonary disease (COPD) for more than ten years, and underwent subtotal gastrectomy due to gastric cancer in 2004. He did not have bronchial asthma and was not allergic to any agents. His renal function was normal in September 2009. At admission, laboratory tests showed severe renal injury (serum creatinine: 5.0 mg/dl), and a urinalysis showed significant proteinuria (3.1 g/gCr), hematuria, and red blood cell casts. His diagnosis was rapidly progressive glomerulonephritis. The other laboratory data included a white blood cell count of 4,600 μl^−1^, eosinocyte count of 78 μl^−1^, hemoglobin of 7.9 g/dl, serum albumin of 3.18 g/dl, CRP of 2.0 mg/dl, and normal transaminase levels. The C3 and C4 levels were normal at 77 and 33 mg/dl, respectively. The serologic evaluation was negative for antinuclear antibody, myeloperoxidase-ANCA, and anti-glomerular basement membrane antibody. His serum PR3-ANCA level was elevated to 330 U/ml. His blood pressure was 132/76 mmHg, he had a regular heartbeat of 84 bpm, and his temperature was 37.8 °C. Cardiac examination revealed no increase in the area of cardiac dullness, and lung and abdominal examinations revealed no abnormalities. Significant leg edema was observed. Pertinent negative examination findings included the absence of purpura, peripheral neuropathy, arthralgia, and ocular inflammation. There was no evidence of infection in the whole-body computed tomography (CT) findings, in any of the cultures, including blood, urine, and sputum, or in a thorough physical examination. A renal biopsy revealed necrotizing and crescentic glomerulonephritis with no granuloma formation (Fig. 1). Unfortunately, the tissue sample was insufficient to perform an immunofluorescence study. However, the histopathological findings were consistent with a diagnosis of ANCA-associated vasculitis. This case did not meet the criteria of granulomatosis with polyangiitis [19], because the chest CT did not show any abnormal chest findings, such as fixed pulmonary infiltrates, nodules, or cavitations, and a proficient otolaryngologist detected no nasal or oral inflammation. The patient was treated with m-PSL at 0.5 g/day for three days, followed by oral PSL at 30 mg/day. As a result of the PSL treatment, the patient’s symptoms, including fever, general fatigue, and appetite loss, disappeared. Because his renal insufficiency appeared to be irreversible regardless of the administration of PSL, outpatient maintenance HD (3 days a week; 3.5-h HD; dry weight, 52 kg; blood flow, 200 ml/min; dialysate flow, 500 ml/min; dialyzer, BG-1.3PQ: polymethyl methacrylate, 1.3 m^2^, Toray, Chiba, Japan; Kt/v, 1.01) was initiated, and the patient was maintained on PSL alone. Although the PR3-ANCA level decreased to 82.1 U/ml in the first two months, the PR3-ANCA level did not decrease further. Instead, it gradually increased after March 2011. After the PSL was tapered to 5 mg/day, the patient’s PR3-ANCA and CRP levels were elevated to 118 U/ml and 13.8 mg/dl, respectively (Fig. 2).

**Fig. 3:**
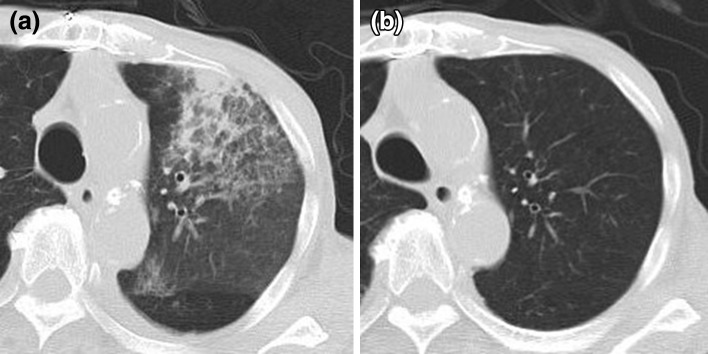
CT scan at the level of the upper lobe in July 2011. **a** Consolidation in the left upper lobe is apparent. **b** The CT scan performed 4 months later at the same level shows resolution of the consolidation

**Fig. 4:**
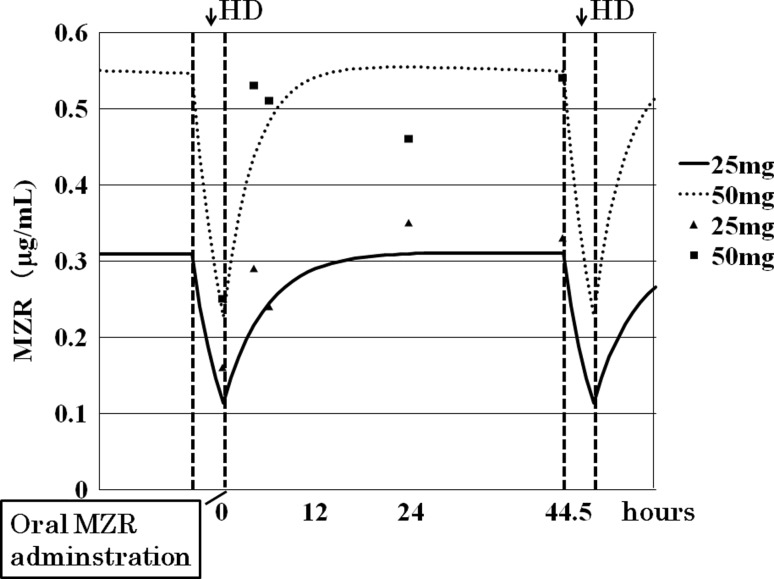
Serum MZR concentrations after the oral administration of 25 and 50 mg of MZR, immediately following dialysis, measured at five time points: immediately after dialysis, 4 h after dialysis, 6 h after dialysis, 24 h after dialysis, and immediately before the next dialysis session (after 44.5 h). The patient’s dialysis conditions, which could influence the serum MZR concentration, were not changed during MZR therapy. The kinetics of the MZR were found to be adequately fitted by a one-compartment model to estimate its serum concentration. Therefore, we drew the kinetic curves using this model, employing higher intradialytic elimination rate constants that were calculated using the semi-log graph

In July 2011, the patient was readmitted because of fever, cough, and progressive dyspnea that had lasted for a week. The chest CT showed consolidation in the left upper lobe (Fig. 3a). He was initially treated with antibiotics, but his symptoms did not improve. Bronchoalveolar lavage fluid (BALF) analysis (lt.B^3^) was then performed. Histopathological analysis of the BALF showed hemosiderin-filled macrophages, confirming that the alveolar hemorrhage was a relapse of the ANCA-associated vasculitis. Taking into consideration that the patient was elderly and on maintenance HD, it was possible that a relatively low dose of steroids would be effective to relieve his symptoms, and that strong immunosuppressive agents such as cyclophosphamide would be harmful rather than beneficial. Therefore, to treat the alveolar hemorrhage, m-PSL at 0.25 g/day was administered for 3 days, followed by oral PSL at 15 mg/day. Steroids relieved his symptoms thereafter. Two weeks later, the patient was given MZR (25 mg) immediately after each HD session as a combination therapy. This treatment decreased the levels of PR3-ANCA and CRP within 3 months. The CT findings also showed resolution of the alveolar hemorrhage (Fig. 3b). The serum concentration of MZR was measured at various time points after each HD session (Fig. 4), and the maintenance dose was set at 50 mg. At his most recent examination, the patient’s serum PR3-ANCA and CRP levels were 8.3 U/ml and 0.1 mg/dl, respectively. The dosage of oral PSL was successfully tapered to 10 mg/day, and the patient achieved and has maintained a state of remission (Fig. 2). The administration of MZR did not cause any drug-related adverse reactions, such as pancytopenia, liver injury, or infection.

## Discussion

Mizoribine is the first imidazole with demonstrated biological activity. It has inhibitory effects on T- and B-lymphocyte activity in vivo, and its pharmacokinetics and immunosuppressive effects have been measured in vivo [4, 5]. In view of its inhibition of purine synthesis and lack of toxicity, MZR has been widely used as a component of immunosuppressive drug regimens in Japan.

MZR (range 0.1–5 μg/ml) is reported to inhibit the human mixed-lymphocyte reaction by 36.4–62.2 %, with 50 % inhibition occurring at a concentration of ~1 μg/ml [9]. Peak MZR blood levels of >0.66 μg/ml are effective for improving the clinical signs and symptoms of lupus nephritis [20, 21]. On the other hand, liver dysfunction and thrombocytopenia can develop at a trough level of >5 μg/ml [9]. Therefore, the blood level of MZR should be monitored to make sure it stays within the safe and optimal therapeutic range.

After oral administration, MZR is absorbed from the gastrointestinal tract and enters cells according to the concentration gradient between the extracellular and intracellular environment. It is completely eliminated from the blood circulation within 24 h, excreted in urine (85 %), feces (9.7 %), and bile (<1 %) [18]. MZR is a dialyzable compound, and its serum concentration is reportedly reduced by 43 % after a single 4 h HD session [22]. As MZR undergoes very little metabolism in the body, the main pathway for its elimination from the body of a patient with end-stage renal failure is thought to be the dialysis session. In our patient, MZR administered immediately after HD sessions was rapidly absorbed, and the blood concentration peaked soon afterwards. Once the MZR distribution in the body reached almost a steady state, its concentration was maintained at about the same level until the next HD session (Fig. 4). Therefore, it seems reasonable to consider that the serum MZR concentration observed immediately before HD is close to* C*_max_ [23]. However, because the peak MZR concentration depends on the residual renal function, even in a patient requiring HD, we measured the precise serum concentration to determine the appropriate dosage of MZR.

In the present case, the* C*_max_ of 25 mg MZR administered orally immediately after HD was 0.35 μg/ml, and that of 50 mg MZR was 0.53 μg/ml (Fig. 4). Although these concentrations were lower than the suggested effective dose [20, 21], the PR3-ANCA titer was still reduced sufficiently to maintain remission of the ANCA-associated vasculitis. We do not know the exact reason for MZR’s effectiveness at the lower concentration; however, it was recently reported that 14-3-3 proteins are MZR-binding proteins [24]. The 14-3-3 proteins interact with many proteins involved in cellular signaling, including the glucocorticoid receptor (GR), and GR’s transcriptional activity is enhanced by its interaction with 14-3-3 proteins. Takahashi et al. [24] showed that MZB affects the conformation of 14-3-3 proteins to enhance their interaction with GR, and MZB dose-dependently enhances 14-3-3η’s interaction with GR in vitro. MZR also enhances the induction of GR activity by dexamethasone [24]. These findings indicate that MZR may elicit its therapeutic effect through the regulation of GR function. MZR can augment a steroid’s effect, thus allowing lower doses of steroids to be used [9, 24]. In addition, Sugiyama [25] reported a case of lupus nephritis in which MZR treatment enabled the glucocorticoid dose to be decreased without disease recurrence. Thus, the combination therapy with MZR may have made it possible to taper the PSL in our patient while maintaining the remission of ANCA-associated vasculitis, even at a low MZR concentration.

MZR in combination with PSL seems to be well tolerated and effective as a maintenance therapy for ANCA-associated vasculitis, even in a patient requiring HD. In addition, MZR was useful for tapering the PSL dosage in this patient. Long-term treatment with MZR for this disease is attractive because of this drug’s effectiveness and low toxicity.
